# Zinsser-Cole-Engmann syndrome: A rare case report with literature review

**DOI:** 10.4317/jced.51274

**Published:** 2014-07-01

**Authors:** Altaf H. Chalkoo, Vibhuti Kaul, Lateef A. Wani

**Affiliations:** 1 Professor and Head of Department. Department of Oral Medicine and Radiology, Government Dental College and Hospital, Srinagar; 2 Post Graduate Student. Department of Oral Medicine and Radiology, Government Dental College and Hospital, Srinagar; 3 Assistant Professor. Department of General Pathology, Government Medical College, Srinagar

## Abstract

Zinsser-Cole-Engmann syndrome, more commonly known as Dyskeratosis Congenita, is a heritable genodermatosis having an estimated incidence of 1 in 1 million people. It is important for an oral physician to be aware of this condition as oral leukoplakia occurs in this condition as part of a classic triad along with reticulate skin pigmentation and nail dystrophy. Besides these, there may be myriad multisystem involvement as well. These individuals have a high predilection for developing malignancies as well as other grave life-threatening conditions. Timely diagnosis and management of these cases may help improve their morbidity and mortality, for which oral physicians can play a major role in recognizing the cases. This will only be possible when more of such cases are reported in dental literature. Here we present a case report of a 30 year old male patient who reported to our department with all the characteristic features of the triad and a few additional findings concordant to the disease as well.

** Key words:**Zinsser-Cole-Engmann syndrome, Dyskeratosis Congenita, leukoplakia, genodermatosis, skin pigmentation, nail dystrophy, progeria, hematological disturbances.

## Introduction

Detection of leukoplakia is a relatively common finding in day to day dental practice. However, its occurrence as a part of a clinical syndrome is very rare. One such condition is Dyskeratosis congenita [DC]. It is a heritable genodermatosis, which was first described in 1906 by Zinsser (1). Later Engmann and Cole reported other cases in detail and hence it is also known as Cole-Engmann syndrome or Zinsser-Cole-Engmann syndrome. Classically, described as a triad of skin pigmentation, nail dystrophy and oral leukoplakia (2). DC is a fatal condition in which majority of the patients develop aplastic anemia and malignant transformation occur in the keratotic white patches which is of considerable interest to an oral physician. Estimated incidence is 1 in 1 million people. DC is genetically heterogeneous, with X-linked recessive, autosomal dominant and autosomal recessive subtypes but majority of the cases show X-linked recessive inheritance. Because of this type of inheritance pattern, the ratio of men to women affected by the disorder is approximately 13:1. DC is most commonly caused by a mutated gene, DKC1, located on the X chromosome. The mucocutaneous features of DC typically develop between ages 5 and 15 years. Here we present a case report of one such patient reporting to our department exhibiting the classic triad sans the hematological dysfunction.

## Case Report

**Figure 1:**
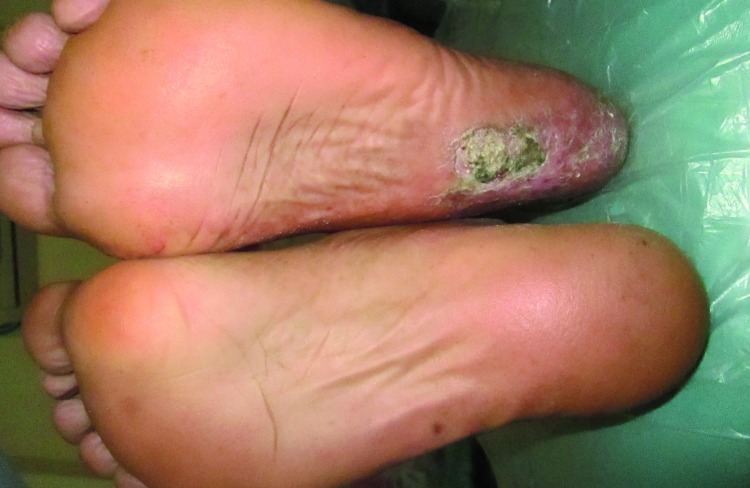
Plantar keratosis.

**Figure 2:**
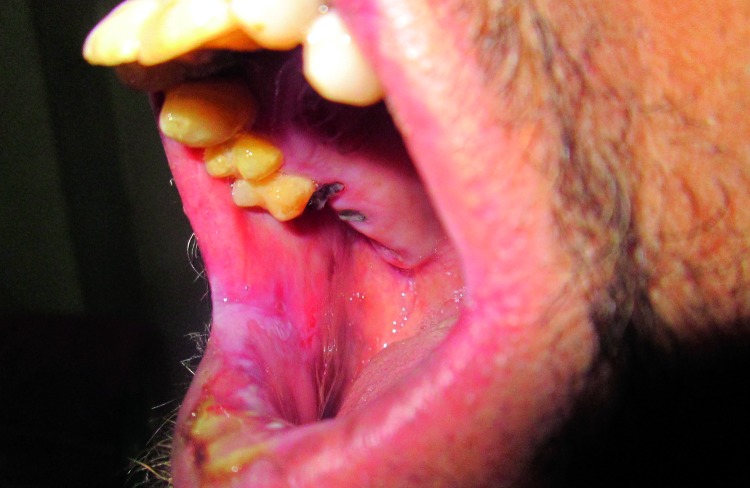
Leukoplakia near the right commissure region on buccal mucosa.

A 30 year old, unmarried, illiterate, unemployed male reported to the Department of Oral Medicine and Radiology, Government Dental College, Srinagar, with a chief complaint of ulcer on the lower lip. History and examination revealed various findings present since birth in the patient. General physical showed a normal intelligence with premature graying of hair, primary poikiloderma with triad of skin atrophy, pigmentations and telangiectasias especially on the dorsum of hands, nail dystrophy with longitudinal splitting in one finger nail, adermatoglyphia [loss of dermal ridges on fingers and toes], plantar keratosis (Fig. 1), photophobia, epiphora [due to lacrimal stenosis], loss of eye lashes. Patient had progeria-like facies and mandibular retrognathism. On Intra-oral examination, ulcers were seen on the lower lip due to trauma from the upper centrals impinging on them. Homogenous leukoplakia of right buccal mucosa near commisure region was detected involving an area of approx. 2x1 cm. (Fig. 2) There was generalized periodontitis and the patient had multiple carious and missing teeth. Lateral skull radiograph showed presence of intracranial calcifications.

Considering the symptoms of skin pigmentation, nail dystrophy and white patch a provisional diagnosis of DC was made. Hematological investigations were carried out to detect any concomitant aplastic anaemia but the values were within normal limits. Family history was positive for brother, who passed away due to same condition at a very young age. Pedigree analysis suggested Mendelian X-linked recessive pattern of inheritance. He gave a medical history of undergoing treatment for the dermal lesions. He had a history of burning micturition, headaches and showed signs of depression. There was no history of prior hospitalizations. Personal history revealed history of smoking 1 pack of cigarettes per day since last 15 years. Patient was in a habit of taking a bland diet due to burning sensation.

**Figure 3:**
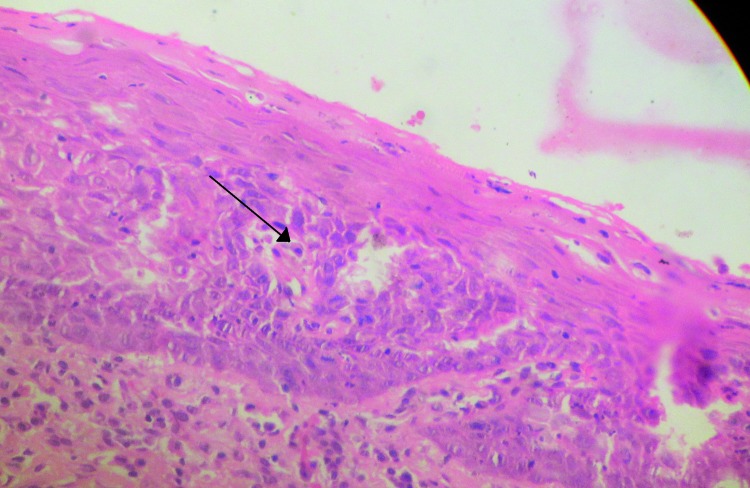
Immature keratinization seen in deeper layers (arrow).

Excisional biopsy of the oral mucosal lesion was taken. Histopathological report confirmed the clinical diagnosis of leukoplakia with areas of epithelium showing moderate to severe hyperparakeratosis, acanthosis of the spinous layer with submucosa showing dense mixed inflammation and vascularization. Some areas of moderate dysplasia were also seen with increased Nuclear:Cytoplasmic ratio, pleomorphic nuclei, immature keratinization (Fig. 3) and abnormal mitoses.

Follow-up was done at 1 week, sutures were removed, however the surgical site showed relatively delayed wound healing which is attributable to the condition. Patient was prescribed a daily supplement of oral antioxidants which included lycopenes, green tea extracts, wheat germ oil, calcium ascorbate, selenium dioxide and zinc sulphate. A diet chart was formulated specifically designed for the patient to meet his requirements. Counseling was done for smoking habit cessation to which the patient has responded well, being tobacco-free for over a year now. He was taught self-examination of the oral cavity to be conducted at home along with meticulous oral hygiene maintenance. Since then, the patient reports for regular recalls every 3 months to our department and over the past one and a half year, there has been no recurrence of the lesion or appearance of new lesions.

## Discussion

Dyskeratosis congenita [DC], an inherited syndrome, first described by Zinsser in 1910, is characterized by the triad of reticulated skin pigmentation, nail dystrophy [both occurring in 100% of cases], and white plaques [80% of cases]; typically occurring in the oral cavity. Other features occur with lower frequencies and involve virtually every organ system (3). DC is related to telomerase dysfunction; all genes associated with this syndrome [DKC1, TERC, TERT, TINF2 and NOP10] encode proteins in the telomerase complex responsible for maintaining telomeres at the ends of chromosomes. Patients with DC have reduced telomerase activity and abnormally short tracts of telomeric DNA compared with normal controls.

Oral manifestations of DC include leukoplakia most commonly affecting the tongue, followed by the buccal mucosa (4). Lichen planus or lichenoid lesions instead of leukoplakia have also been reported (5). Besides these, severed periodontal destruction especially juvenile periodontitis has been reported which might be due to associated neutropenia (6). Increased incidence of dental caries has also been seen in such patients which has been postulated to be due to defects in enamel calcification (5).

Cannel H [1971] postulated the nature and changes in the oral manifestations of this condition in relation to time:

• Age 5-14 years- White patches of necrotic epithelium or possible candidal infections. Patches always preceded

by vesicles and ulcerations.

• Age 14-20 years- Recurrent ulcerations and erythroplakia.

• Age 2-30 years- Erosive leukoplakia and carcinoma.

Patients suffering from DC have been reported to have a markedly increased incidence of malignancies (8). These include mucosal neoplasms, particularly squamous cell carcinoma of the mouth, nasopharynx, esophagus, rectum, vagina, or cervix. The development of Hogkin’s disease and adenocarcinoma have been recorded (9).

The question of ‘Why such a high incidence in DC patients?’ lead to Electron microscopy studies revealing that cells in dyskeratosis congenita have an embryonic immature nucleus, which have higher chances to undergo malignant transformation (8). In addition, barrier zone of epithelium is less effective in dyskeratosis congenita than the normal epithelium causing increased permeability of noxious substances and carcinogens to the germinal layers (10).

The main causes of death are Bone marrow failure/immunodeficiency [~60-70%], pulmonary complications [~10-15%] and malignancy [~5-10%] (11).

There is no effective and curative treatment for DC. Some interceptive, preventive and palliative measures can be adopted for which an early diagnosis is essential. Patient should be kept under observation and recalled for periodic follow up. In follow-up visits biopsy and complete blood picture should be advised to detect any malignant changes in white patches or developing haematopoietic disorder.

Chemotherapy with bleomycin or cyclophosphamide is effective for treating leukoplakia associated with DC (6). Tomita and Ishii reported that a vitamin A derivative, etretinate is effective for treating leukoplakia in DC (12). However, side effects like exfoliative cheilitis, angular cheilitis and teratogenicity can occur. Excisional biopsy can also be indicated to eliminate the possibility of development of carcinoma in leukoplakic areas. For extensive lesions in which surgery is not possible administration of steroids and testosterone can be used (6).

Use of the anabolic steroid oxymetholone and haematopoietic growth factors such as erythropoietin [epoetin alpha], granulocyte macrophage colony stimulating factor and granulocyte colony stimulating factor [filgrastim] can produce improvement in the haematopoietic function (13). The only long term cure for the haemopoietic abnormalities is allogeneic haematopoietic stem cell transplantation, but this is not without risk. There is still significant mortality associated with bone marrow transplants for DC patients when compared with other bone marrow failure syndromes. One of the main reasons for this is the high level of pulmonary/vascular complications that present in these patients probably as a result of the underlying telomere defect. In future gene therapy can provide an alternative therapy for management of this fatal condition. Nonmyeloablative hematopoietic SCT conditioning regimens [i.e., reduced-intensity conditioning] with fludarabine may offer better outcomes. A 2007 review showed a 22% mortality rate with reduced-intensity conditioning in DC treatment versus a 71% mortality rate with traditional myeloablative regimens. Treatment regimes exploring the combination of fludarabine, cyclophosphamide and anti-thymocyte globulin, are being explored (14).

We conclude that a long-standing history of a white patch in the mouth in a young patient, with or without significant history of tobacco usage, occurring concomitantly with mucocutaneous and systemic features as described should invoke a suspicion of a heritable genodermatosis such as Dyskeratosis congenita. Such patient have a high predilection for malignancies and an oral physician can play a pivotal role in detecting and managing such cases as a part of a multidisciplinary team.
